# Flow-cytometric profiling of large extracellular vesicles as immunophenotypic biomarkers in head and neck squamous cell carcinoma

**DOI:** 10.1007/s00262-026-04426-8

**Published:** 2026-05-27

**Authors:** Judith Büntzel, Markus Maulhardt, Alexander Casimir Angleitner, Matthias Schulz, Daniel Köberle, Said Naser, Clara Zwerenz, Charlotta Friederike Pagel, Andrea Hille, Anna-Carina Hund, Jens Büntzel

**Affiliations:** 1 Department of Hematology and Medical Oncology, University Medical Center Goettingen, 37075 Göttingen, Germany; 2 Department of Radiation Therapy and Radiation Oncology, University Medical Center Goettingen, 37075 Göttingen, Germany; 3 Department of Personalized Oncology, University Hospital Mannheim, and Medical Faculty Mannheim, University of Heidelberg, 68167 Mannheim, Germany; 4 Department of Otolaryngology, Head Neck Surgery, Südharz Hospital, 99734 Nordhausen, Germany

**Keywords:** Head and neck cancer, NSCLC, Large extracellular vesicles, Liquid biopsy, Flow cytometry, Biomarker profiling

## Abstract

**Background:**

Large extracellular vesicles (large EV) released from tumor and benign cells are detectable in blood and hold potential as non-invasive biomarkers. While their diagnostic relevance has been shown in several malignancies, their role in head and neck squamous cell carcinoma (HNSCC) remains insufficiently characterized.

**Methods:**

Large EV were isolated from peripheral blood of patients with HNSCC (n = 66), non-small cell lung cancer (NSCLC; n = 52; adenocarcinoma n = 39, squamous cell carcinoma (SQCCL) n = 11), and healthy controls (n = 11) by differential centrifugation. Flow cytometry quantified surface expression of EGFR, EPCAM, MUC1, and PD-L1. Associations with clinical parameters were analyzed using t-tests, Spearman correlations, logistic regression, and random forest modeling.

**Results:**

HNSCC-derived large EV showed significantly higher EGFR and MUC1, but lower EPCAM expression compared to controls. PD-L1 expression increased with advancing tumor stage and was positively associated with metastatic status, whereas EGFR levels declined in metastatic disease. Combined ROC analysis of EGFR, EPCAM, and PD-L1 yielded an AUC of 0.785 (*p* = 0.003), distinguishing HNSCC from controls. Comparative profiling revealed higher EGFR and EPCAM expression in HNSCC versus NSCLC, while MUC1 predominated in NSCLC, particularly in SQCCL; notably, the NSCLC cohort was predominantly adenocarcinoma. A marker panel comprising EGFR, EPCAM, and MUC1 differentiated HNSCC from SQCCL in this limited subgroup (n = 11) with 96.97% sensitivity, 45.45% specificity, 92.75% positive predictive value and 75.00% negative predictive value.

**Conclusion:**

Flow-cytometric profiling of circulating large EV provides a feasible liquid biopsy approach for tumor characterization in HNSCC. PD-L1 expression reflects tumor burden, and combined large EV marker analysis enables differentiation between HNSCC and primary squamous lung carcinoma, supporting its diagnostic utility in clinical oncology.

**Supplementary Information:**

The online version contains supplementary material available at 10.1007/s00262-026-04426-8.

## Introduction

Head and neck squamous cell carcinoma (HNSCC) ranks among the six most common malignancies worldwide [1]. In Germany, approximately 4560 women and 12,600 men were diagnosed with head and neck cancer in 2014 [2]. Major etiological factors include prolonged tobacco and alcohol consumption, as well as infections with oncogenic viruses such as human papillomavirus (HPV) and Epstein–Barr virus (EBV) [3]. Beyond these established carcinogens, immune evasion has emerged as a central hallmark of HNSCC, with tumor expression of immune checkpoint molecules—particularly programmed death ligand 1 (PD-L1)—contributing to suppression of antitumor T-cell responses and resistance to therapy [4]. Due to the frequent origin of lesions from deep anatomical sites within the larynx, pharynx, and the oral or nasal cavity, HNSCC is often diagnosed at an advanced stage [5]. Despite considerable research efforts, no reliable biomarker for the early detection or prognosis of HNSCC has been established to date [6].

In recent years, the application of liquid biopsy approaches has gained attention for their potential to enable real-time molecular and immunological monitoring of cancer; however, a lack of methodological standardization continues to limit their clinical implementation [6]. Among circulating analytes, large extracellular vesicles (large EV), defined here as plasma membrane–derived extracellular vesicles ranging from approximately 100–1000 nm in diameter, represent a biologically active source of biomarkers [7]. In addition to carrying tumor-associated molecules such as EGFR and MUC1, large EV can transfer immune checkpoint proteins, including PD-L1, thereby modulating antitumor immune responses and contributing to systemic immune escape [8]. These vesicles are detectable in multiple body fluids, including saliva, urine, and blood, offering minimally invasive access [7] to both tumor and immune-derived signals.

Previous studies have demonstrated that elevated levels of large EV in plasma and saliva correlate with tumor burden and lymph node metastasis in patients with oral cavity tumors [9]. Additionally, small extracellular vesicles (sEV) derived from patients exhibited distinct infrared signatures compared to healthy controls [10], and higher concentrations of sEV were associated with disease recurrence [11]. Although total large EV abundance is increased in HNSCC patients [12], quantitative assessment alone lacks diagnostic specificity. Recent research has therefore shifted towards characterizing the molecular and immunological cargo of vesicles, including PD-L1, EGFR, and CD44, which are mainly studied in sEVs [5]. Beyond HNSCC, large EVs have also been investigated as circulating biomarkers in other solid malignancies, including NSCLC [13], hepatobiliary cancers [14], prostate cancer [15], and clear-cell renal cell carcinoma [16], supporting their broader translational relevance across tumor entities. These findings highlight the potential of EV-bound immune checkpoint molecules as dynamic biomarkers reflecting tumor progression and immune escape.

In this proof-of-concept study, we applied a translational immuno-oncology approach to evaluate whether large EV could serve as clinically accessible biomarkers in head and neck squamous cell carcinoma (HNSCC). Unlike most prior work focusing on sEVs, we selected large EVs for their straightforward isolation and direct suitability for flow-cytometric immunophenotyping [17]. Using a pre-established panel of surface markers including the epithelial/tumor-associated markers EGFR, EPCAM, and MUC1 [12] together with the immune checkpoint marker PD-L1, we investigated whether large EV profiling could discriminate HNSCC from healthy individuals and from patients with non-small cell lung cancer (NSCLC), given the clinically relevant challenge that pulmonary nodules in patients with HNSCC may represent either metastatic disease or a second primary lung malignancy.

## Material and methods

### Patient recruitment and EV isolation

Patients diagnosed with either HNSCC or NSCLC were recruited between June 2016 and February 2024. Control subjects were enrolled between May 2020 and February 2024. Healthy controls were recruited under strict criteria (no history of malignancy and no relevant acute/chronic inflammatory or systemic disease at the time of blood draw). As a consequence, the control cohort was younger than the cancer cohorts (Tables 1 and 3).The study was approved by the local Ethics Committee (approval number 3/2/14, Ethics Committee of the University Medical Center Göttingen), and written informed consent was obtained from all participants. Peripheral blood (15 mL) was collected prior to initiation of any therapy to minimize the influence of apoptotic bodies on vesicle isolation. Clinical information including cancer type, pathological findings, TNM stage, p16 status, PD-L1 expression, and follow-up data was retrieved from patient records. Missing p16 and tissue PD-L1 values reflect routine clinical testing patterns (p16 primarily assessed in oral cavity/oropharynx; tissue PD-L1 mainly evaluated in advanced-stage cases) and retrospective record availability. Large EVs were isolated from plasma by differential centrifugation as previously described [18]: Peripheral blood was collected into EDTA-containing tubes and processed within 2h after withdrawal. Plasma was obtained by centrifugation at 1200 × g for 15 min at room temperature, followed by a second centrifugation step at 1500 × g for 15 min to remove residual blood cells, larger debris, and platelets. The resulting supernatant was transferred to centrifugation tubes and large EV were pelleted by centrifugation at 14,000 × g for 35 min at 4 °C. After removal of the supernatant, the pellet was washed once in PBS and centrifuged again at 14,000 × g for 35 min at 4 °C. The final large EV pellet was resuspended in PBS (PBS; Gibco, Thermo Fisher Scientific, Waltham, MA, USA) and stored at − 20 °C until further analysis.

**Table 1:** Clinical characteristics HNSCC cancer patients and controls

HNSCC patients		N [cases]
Total		66
Age [years] median (min–max)		64.63 (37.18 – 84.56)
Sex	Male	52 (79%)
	Female	14 (21%)
Survival status at last follow-up	Alive	43 (65%)
	Deceased	23 (35%)
Localization	Oral cavity	12 (18%)
	Oropharynx	25 (38%)
	Hypopharynx	6 (9%)
	Larynx	15 (23%)
	Sinunasal	3 (5%)
	Salivary glands	4 (6%)
	others	1 (2%)
T-Status	T1	7 (11%)
	T2	14 (21%)
	T3	20 (30%)
	T4	20 (30%)
	n/a	5 (8%)
N-Status	N positive	35 (53%)
	N negative	26 (39%)
	n/a	5 (8%)
M-Status	M0	50 (76%)
	M1	15 (23%)
	n/a	1 (2%)
Extranodal spread	Positive	18 (27%)
	Negative	26 (39%)
	n/a	22 (33%)
Grading	1	1 (2%)
	2	38 (58%)
	3	13 (20%)
	n/a	14 (21%)
p16-status	Positive	16 (24%)
	Negative	13 (20%)
	n/a	37 (56%)
PD-L1-status (tissue)	CPS ≥ 20	11 (17%)
	1 ≤ CPS < 20	11 (17%)
	CPS < 1	3 (5%)
	n/a	41 (67%)
Healthy controls		
Age [years] median (min–max)		31 (24–54)
Sex	Male	5 (45%)
	*Female*	6 (55%)

### Western blot

Protein expression in cell lysates, human plasma, and patient-derived large EVs was analyzed by immunoblotting. The following primary antibodies were used: α-ACTININ-4 (sc-390205), RGAP1 (sc-271110), β-ACTIN (sc-47778), and APOA1 (sc-376818) (all Santa Cruz Biotechnology). Horseradish peroxidase (HRP)-conjugated secondary antibodies were obtained from Cell Signaling Technology (#7076). Equal amounts of protein (17 µg per lane) were separated by SDS-PAGE and transferred to nitrocellulose membranes. After blocking with TBST buffer (137 mM NaCl, 20 mM Tris, pH 7.6, 0.1% Tween-20), membranes were incubated with the respective primary antibodies overnight at 4 °C. Following three washes in TBST, HRP-linked secondary antibodies were applied for 1h at room temperature and membranes were washed again three times. Chemiluminescent signals were visualized using ECL Prime (GE Healthcare, Chicago, IL, USA) on a LAS-4000 imaging system (Fujifilm, Düsseldorf, Germany). Ponceau S staining served as a routine loading control.

### Flow cytometry

For surface marker characterization, large EV were stained with the following fluorescently labeled antibodies: anti-MUC1 (clone 16A, #355604, BD Biosciences, Franklin Lakes, NJ, USA), anti-EGFR (clone AY13, #352904, BD Biosciences), anti-EpCAM (clone 9C4, #324208, BD Biosciences) and anti-PD-L1 (clone MIH3, #374510, BD Biosciences). For flow-cytometric analysis, large EV preparations were normalized by total protein content, and 5 µg protein was used per staining reaction. Total protein was used as a surrogate measure of vesicle input, consistent with our previously established methodological approach [18]. Thus, all flow-cytometric measurements were performed using equal large EV input. Briefly, 5 µg of large EV were first blocked with 20 µL of PBS containing 1% vesicle-depleted fetal calf serum (FCS; Gibco, Thermo Fisher Scientific) for 30 min at room temperature (RT). Samples were then incubated with the corresponding antibodies for 20 min at RT. Unstained large EV preparations were included as negative controls, and corresponding isotype control antibodies were used at matching concentrations to assess background fluorescence and nonspecific binding. After staining, 200 µL of PBS were added before acquisition. Flow cytometric analysis was performed using a FACS Canto II flow cytometer (BD Biosciences), and data were analyzed with FACS Diva software (version 9.0.1, BD Biosciences) and FlowJo software (version 10.8.1; Treestar, now part of BD Biosciences; https://www.flowjo.com/). Instrument thresholds were reduced to the lowest possible setting, and lEVs were identified on forward scatter (FSC) versus side scatter (SSC) plots in logarithmic scale. The resulting lEV gate was subsequently used to evaluate marker-specific fluorescence signals in the corresponding channels as previously described [12].

### Statistical analysis

For statistical analysis, all marker data were log2-transformed and Pareto-scaled. Thus, data processing included both experimental normalization of large EV input prior to staining (equal protein amount per sample, used as a surrogate of vesicle input [18]) and subsequent statistical normalization of marker intensities for comparative analyses. All statistical analyses were conducted using GraphPad Prism (version 9.0; GraphPad Software, La Jolla, CA, USA). Welch’s ANOVA, Welch’s t-test, and Spearman’s rank correlation coefficient (Spearman’s r) were employed to assess associations between large EV biomarker expression patterns and clinical characteristics. Multiple logistic regression analysis was used to generate receiver operating characteristic (ROC) curves for combined biomarker evaluation. Principal component analysis (PCA), heatmaps, and random forest classification were performed using MetaboAnalyst (version 6.0; Xia Lab, McGill University, Montréal, QC, Canada; [19]). Heatmaps were generated from log2-transformed and Pareto-scaled marker measurement data using Euclidean distance and Ward clustering. Group separation in PCA was assessed by PERMANOVA with 999 permutations. Text refinement and stylistic harmonization were performed with the assistance of ChatGPT-5 (OpenAI, 2025), a large language model used for scientific language editing.

## Results

### Clinical characteristics

A total of 66 samples from patients diagnosed with head and neck squamous cell carcinoma (HNSCC) were included in the analysis. The majority of cases originated from the oropharynx (25/66), larynx (15/66), or oral cavity (12/66). P16 positivity was observed in 18 out of 44 patients, and 15 of 65 patients presented with metastatic disease at the time of inclusion. Detailed clinical characteristics are summarized in Table 1. The mean age of healthy controls was 31 (24–54) years. Healthy controls were considerably younger than HNSCC patients (64.63 (37.18–84.56) years; Table 1), which should be considered when interpreting group comparisons of large EV marker expression. A total of 6 female and 5 male control samples were included.

### Large EV from HNSCC patients exhibit a distinct biomarker expression profile

**Fig. 1:**
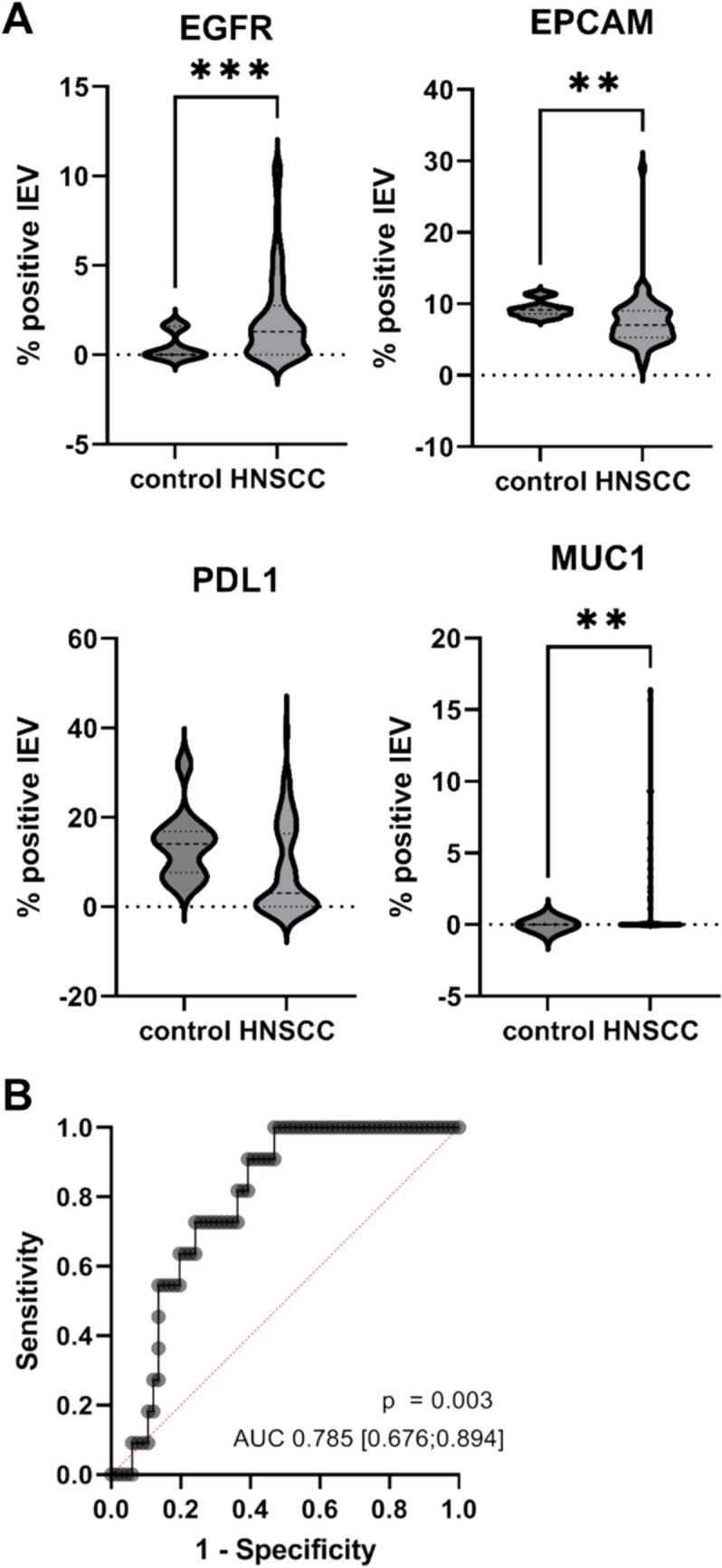
large EV profile of head neck cancer patients (HNSCC) differs from healthy controls*.* Controls express less EGFR (*p* < 0.001) or MUC1 (*p* < 0.01) than HNSCC signal in flow cytometry, while EPCAM (*p* < 0.01) is elevated in controls, Welch’s unpaired t-test. **B** Multiple logistic regression, Receiver Operator Curve analysis of PD-L1, EGFR and EPCAM expression is able to differentiate between HNSCC and controls, however specifity is poor

**Fig. 2:**
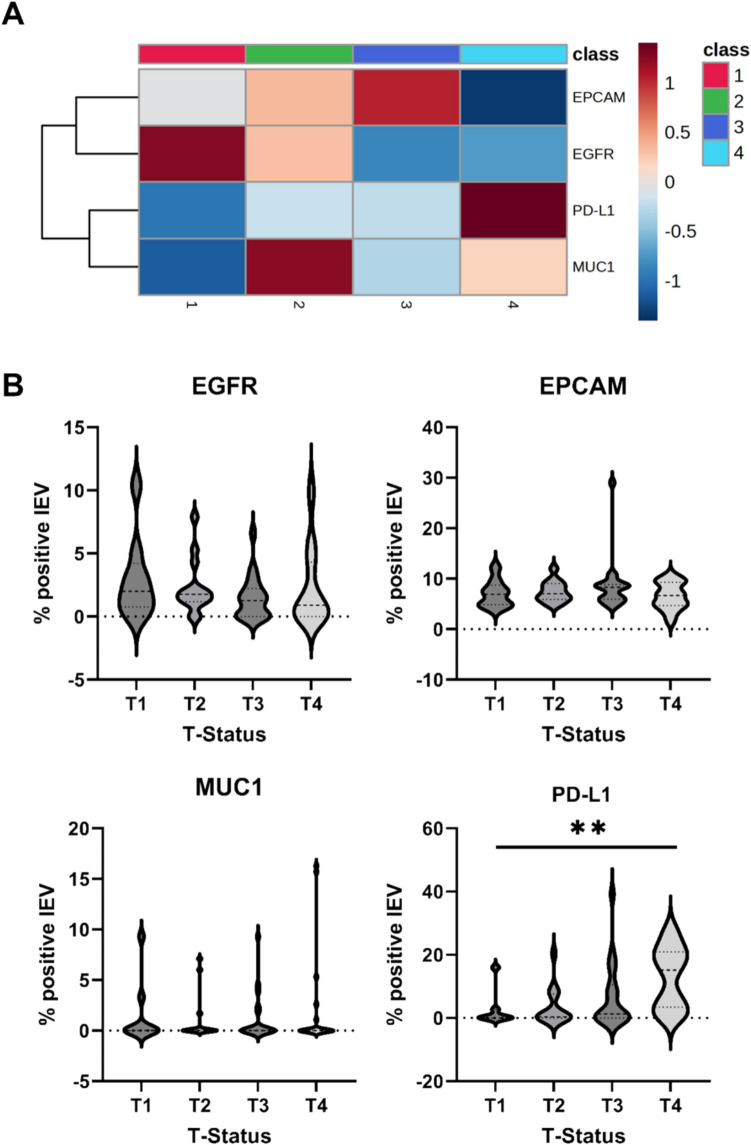
PD-L1 status is associated with increased tumor burden. **A** Heat map of log2-transformed and Pareto-scaled large EV marker measurement data, generated using Euclidean distance and Ward clustering, revealing high PD-L1 expression in patients with T4-stage tumors and high EGFR expression in patients with T1-stage tumors. Group averages are shown. **B** PD-L1 signal on large EV is increased in patients with higher tumour burden (T-status). One-way Anova analysis, flow cytometry

Representative Western blot analysis of isolated large-EV preparations showed the presence of vesicle-associated proteins (α-Actinin-4, RGAP1, and β-Actin), as shown in Supplementary Figure S1. Large EV isolated from HNSCC patients and healthy controls were characterized by flow cytometry. Initial analysis revealed only a trend towards higher PD-L1 expression in healthy control samples (*p* = 0.05, Welch’s unpaired t-test; Fig. 1A). However, PD-L1 expression significantly increased with increasing T-stage (*p* < 0.01, one-way ANOVA; Fig. 2B). When comparing only T1–T3 HNSCC patients to healthy controls, PD-L1 expression was significantly lower in the patient group (*p* < 0.01). Additionally, EPCAM expression was significantly higher in control large EV (*p* < 0.01), whereas EGFR and MUC1 expression levels were elevated in HNSCC samples (*p*_EGFR_ < 0.001, *p*_MUC1_ < 0.01, Welch’s unpaired t-test; Fig. 1A). Multiple regression analysis incorporating EGFR, EPCAM, and PD-L1 expression successfully distinguished HNSCC patients from healthy controls (Fig. 1B). Importantly, the AUC of 0.785 indicates that the combined marker panel captures discriminatory information in this cohort; however, the low specificity at the selected operating point limits its suitability as a standalone clinical classifier. MUC1 was not included in this multivariable model because its distribution in the HNSCC-versus-control comparison did not allow stable parameter estimation in standard logistic regression.

### PD-L1 expression on large EV correlates with tumor burden

To investigate the association between large EV biomarker expression and tumor burden, heat map analysis was performed. This revealed an increase in PD-L1 expression predominantly among patients with T4 tumors (Fig. 2A), whereas EGFR expression was highest in T1-stage tumors. ANOVA confirmed the significant increase of PD-L1 expression with advancing T-stage (Fig. 2B). Furthermore, Spearman correlation analysis demonstrated a positive association between increasing T-stage and the presence of metastasis (Table 2). Neither tumor grading nor nodal status showed significant associations with EGFR, EPCAM, MUC1, or PD-L1 expression (Table 2). Notably, EGFR expression inversely correlated with M-status, consistent with the observation of elevated EGFR expression in patients with low tumor burden (T1-stage) on heat map analysis (Fig. 2A). Additionally, extranodal extension status exhibited a negative correlation with MUC1 expression (Table 2).

**Table 2:** Correlation analysis of HNSCC tumor characteristic’s and large EV signal (flow cytometry)

	Spearman’s r	*p*-value
T-status–PD-L1 large EV	0.3813	0.0022
T-status–MUC1 large EV	− 0.0610	0.6346
T-status–EGFR large EV	− 0.1412	0.2698
T-status–EPCAM large EV	− 0.0488	0.7042
N-status–PD-L1 large EV	0.1335	0.3050
N-status–MUC1 large EV	− 0.1711	0.1875
N-status–EGFR large EV	− 0.0845	0.5180
N- status EPCAM large EV	0.0838	0.5208
M-status–PD-L1 large EV	0.3746	0.0020
M-status–MUC1 large EV	− 0.0871	0.4868
M-status–EGFR large EV	− 0.2436	0.0487
M-status–EPCAM large EV	− 0.0397	0.7507
ENE status–PD-L1 large EV	0.0319	0.8373
ENE status–MUC1 large EV	− 0.3059	0.0435
ENE status–EGFR large EV	0.0403	0.7953
ENE status–EPCAM large EV	0.0237	0.8788
Grading–PD-L1 large EV	0.1496	0.2899
Grading–MUC1 large EV	0.0046	0.9744
Grading – EGFR large EV	− 0.0403	0.7765
Grading–EPCAM large EV	0.0713	0.6156

### Differential expression profiles of HNSCC and NSCLC patients

**Fig. 3:**
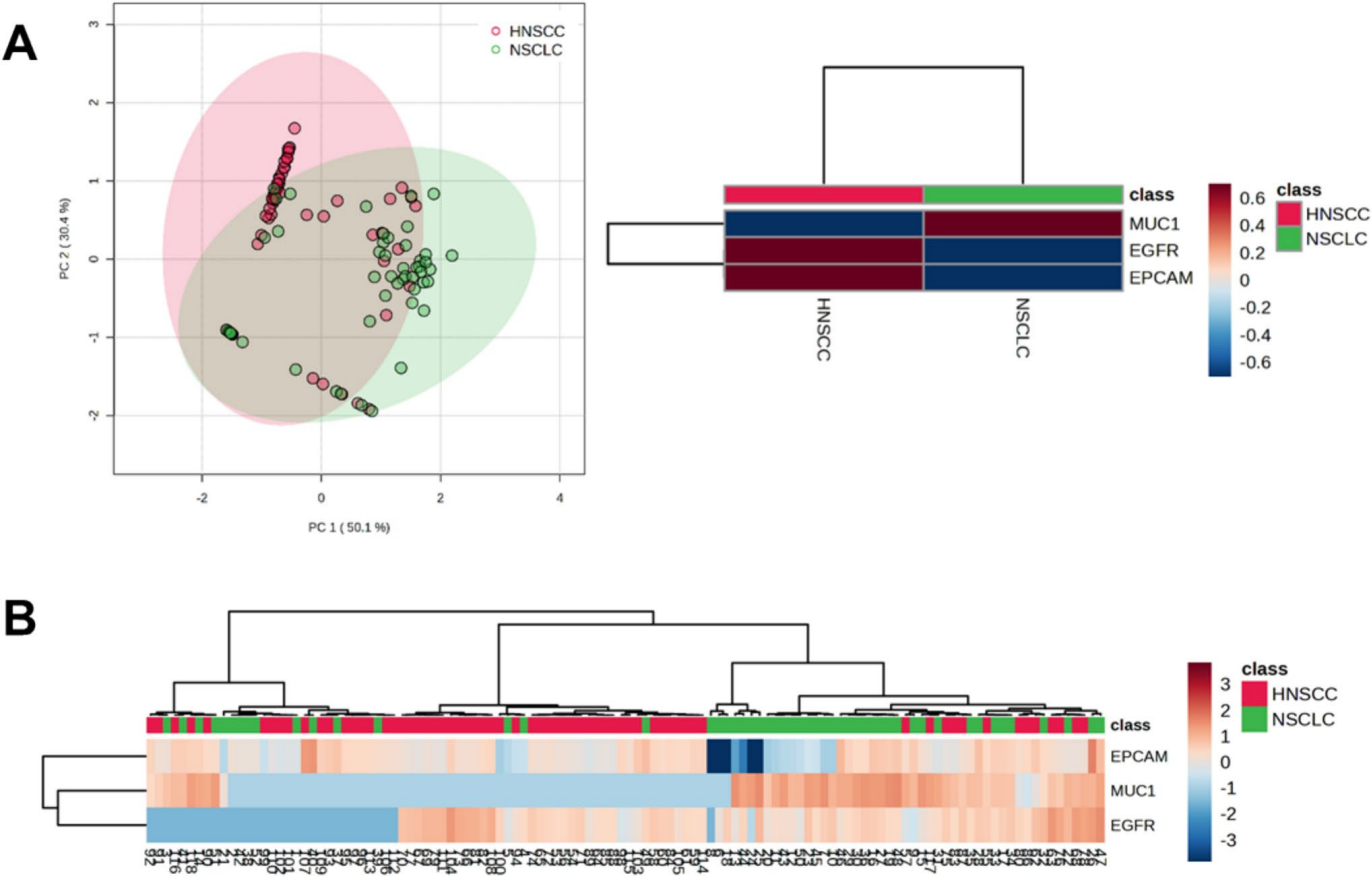
HNSCC and NSCLC patients show different expression patterns of EGFR, EPCAM and MUC1. **A** Principal component analysis (PCA) of log2-transformed and Pareto-scaled marker measurement data revealed partial separation between HNSCC and NSCLC samples; group separation was supported by PERMANOVA (F = 21.443, R^2^ = 0.156, *p* = 0.001; 999 permutations). Heat map analysis of group averages, generated using Euclidean distance and Ward clustering, showed predominantly higher EGFR and EPCAM expression in HNSCC, whereas NSCLC samples were characterized by higher MUC1 expression. **B** Heat map showing case-by-case marker expression patterns based on log2-transformed and Pareto-scaled flow-cytometric measurement data using Euclidean distance and Ward clustering

For comparative profiling between HNSCC and NSCLC, analyses focused on the previously established epithelial/tumor-associated marker panel EGFR, EPCAM, and MUC1. To determine whether the observed expression pattern was specific to HNSCC, we compared large EV profiles from 66 HNSCC patients and 52 NSCLC patients (clinical details provided in Table 3). PD-L1 was not included in this comparative panel, as it was investigated primarily in the HNSCC cohort with regard to its association with tumor burden. PCA demonstrated partial, but not complete, separation between the two groups (Fig. 3A). As an unsupervised exploratory method, PCA was used to visualize global structure in the marker-expression data rather than to demonstrate complete class separation. This group separation was supported by PERMANOVA (F = 21.443, R^2^ = 0.156, *p* = 0.001; 999 permutations). Heat map analysis revealed higher mean expression levels of EGFR and EPCAM in HNSCC samples, whereas elevated MUC1 expression characterized NSCLC samples (Fig. 3A). However, similar to PCA results, a clear distinction was not uniformly observed when analyzing individual cases (Fig. 3B). Random forest analysis yielded an out-of-bag (OOB) error rate of 0.263, with a classification error of 0.269 for NSCLC and 0.258 for HNSCC samples.

**Table 3:** Clinical characteristics NSCLC cancer patients

N [cases]		
Total		52
Age [years] median (min–max)		61 (50–83)
Sex	Male	29 (56%)
	Female	23 (44%)
Survival status at last follow-up	Alive	30 (58%)
	Deceased	21 (40%)
	N/A	1 (2%)
Entity	Adenocarcinoma	39 (75%)
	Squamous cell carcinoma	11 (21%)
	N/A	2 (4%)
Metastasis	Yes	46 (88%)
	No	4 (8%)
	N/A	2 (4%)

### Discriminating between HNSCC and squamous Cell carcinoma of the lung (SQCCL)

**Fig. 4:**
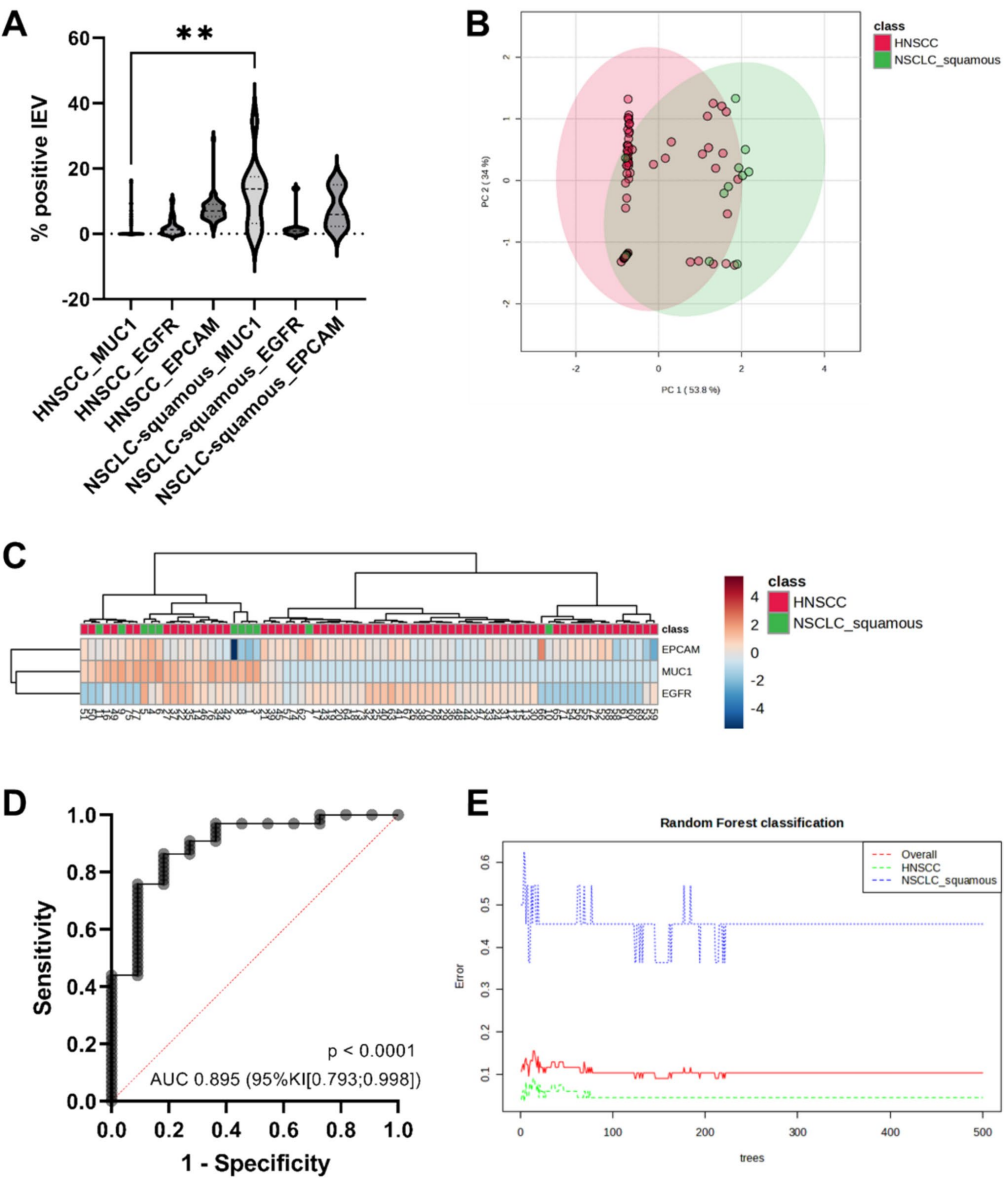
Differentiation between patients with squamous head and neck cancer (HNSCC) and squamous lung cancer (NSCLC_squamous). **A** NSCLC_squamous show a significantly higher expression of MUC1 in flow cytometry (Welch’s unpaired t-test, *p* < 0.01). **B**, **C** Principal component analysis (PCA) and heat map of log2-transformed and Pareto-scaled marker measurement data, generated using Euclidean distance and Ward clustering, showed partial separation between groups. **D** combined Receiver Operator Curve analysis of MUC1, EGFR and EPCAM shows that a differentiation of NSCLC_squamous and HNSCC is possible. Multiple logistic regression, sensitivity 96.97%, specificity 45.45%, positive predictive value 92.75%, negative predictive value 75.00%. **E** Machine learning is able to identify HNSCC patients using expression of MUC1, EGFR and EPCAM. Random forest plot, 500 trees, NSCLC_squamous classification error: 0.455; HNSCC classification error 0.046. *Note*: SQCCL subgroup size was limited (n = 11; Table 3); results should be interpreted as exploratory

While differentiating between HNSCC and NSCLC was promising, we were even more deeply interested in comparing NSCCC and SQCCL samples. Both HNSCC and SQCCL are strongly associated with smoking, and patients with HNSCC are at an increased risk of developing SQCCL [20]. Differentiating between a pulmonary metastasis of HNSCC and a primary SQCCL remains a significant clinical challenge [21]. Therefore, we evaluated whether EGFR, EPCAM, and MUC1 expression profiles could distinguish between these cancer entities. However, the NSCLC cohort was predominantly composed of adenocarcinoma (39/52), whereas only 11 cases represented SQCCL (Table 3). Therefore, all analyses focusing on SQCCL (Fig. 4) should be considered exploratory and interpreted with caution until validated in a larger, histology-balanced cohort. MUC1 expression was significantly higher in SQCCL samples compared to HNSCC samples (Fig. 4A). Subsequent PCA and heat map analyses demonstrated that a subset of HNSCC cases could be clearly separated from SQCCL cases (Fig. 4B–C). Multiple logistic regression using EGFR, EPCAM, and MUC1 achieved a sensitivity of 96.97% and a positive predictive value of 92.75% for identifying HNSCC cases (Fig. 4D). Machine learning via random forest analysis classified HNSCC samples with a low error rate of 0.046, although classification of SQCCL samples remained less accurate (classification error: 0.455; Fig. 4E).

## Discussion

HNSCC is a biologically and immunologically heterogeneous disease. Although PD-L1 and HPV status are routinely assessed to characterize tumor biology and guide therapeutic stratification [22], the availability of reliable immune-related tumor markers for HNSCC remains limited. The diagnostic performance of conventional serum markers such as carcinoembryonic antigen (CEA), squamous cell carcinoma antigen (SCC), tissue polypeptide–specific antigen (TPS), and CYFRA 21-1 remains controversial [23–25]. As a result, recent efforts have focused on circulating analytes such as tumor DNA and microRNA (miRNA). However, despite their promise, neither approach has achieved clinical implementation, largely due to technical variability and insufficient reproducibility [26, 27].

With the exception of certain virus-associated malignancies, such as the monitoring of EBV DNA in nasopharyngeal carcinoma [22, 28, 29], no consensus has been reached regarding the integration of circulating biomarkers into surveillance strategies for HNSCC. While clinical examination and imaging remain the cornerstones of follow-up, these approaches lack sensitivity for detecting minimal residual disease or early recurrence. Consequently, there is a persistent unmet need for circulating immune- and tumor-derived biomarkers that can assist in distinguishing between recurrence, metastasis, and second primary malignancies [22, 30]. This distinction is clinically critical, as metastases are generally managed palliatively [22], whereas second primary cancers may still be curable Pulmonary nodules occur in more than 10% of HNSCC patients [30], rendering lung lesions a frequent and diagnostically challenging finding.

In this exploratory study, we evaluated large EV as a potential platform for immunologically informative biomarker analysis in HNSCC. Because molecular assays such as miRNA or ctDNA analysis remain technically demanding [31] and insufficiently standardized for clinical application [27], we employed flow cytometry, a well-established technique in clinical immunology, to characterize circulating large EV. A previous study by Menck et al. identified distinct large EV surface profiles in cancer patients compared with healthy controls [12]. Building on this work, we quantified the expression of EGFR, EPCAM, MUC1, and PD-L1 on large EV and found significantly higher levels of EGFR and MUC1, but lower EPCAM in HNSCC patients compared with healthy individuals, consistent with prior observations of Menck et al. [12], who likewise described differential expression of epithelial/tumor-associated markers on circulating large EVs/microvesicles in cancer patients relative to healthy controls. Importantly, the present data do not identify a single validated large-EV biomarker for HNSCC. Rather, PD-L1 showed the strongest association with tumor burden, while EGFR, EPCAM, and MUC1 were most informative when interpreted as a combined expression pattern. This is consistent with the fact that the analyzed circulating large-EV fraction represents a heterogeneous vesicle mixture from multiple cellular sources rather than purified tumor-derived vesicles.

Previous studies reported elevated PD-L1–positive extracellular vesicles in cancer patients compared with healthy controls [13]. In contrast, our analysis showed lower PD-L1 expression on large EV from HNSCC patients overall, but a significant increase with advancing tumor stage. Importantly, these comparisons refer to large-EV-associated flow-cytometric PD-L1 signal within the gated circulating large-EV population, not to tumor-cell surface PD-L1 expression or to purified tumor-derived vesicles. This pattern mirrors findings in sEV, where PD-L1 levels rise in advanced HNSCC [32]. One possible explanation for the seemingly lower baseline PD-L1 signal in HNSCC compared with controls is that the flow-cytometrically assessed large EV fraction represents a heterogeneous mixture of vesicles from different cellular sources. In early-stage disease, an increased contribution of tumor-/epithelial-associated large EV that are PD-L1-low could reduce the average PD-L1 signal when analyzing the total large EV gate, whereas with increasing tumor burden the fraction of PD-L1–positive vesicles may rise due to enhanced immune escape mechanisms and/or a higher proportion of tumor-derived PD-L1–positive vesicles. In our cohort, the stage-associated increase was mainly driven by higher PD-L1 levels in advanced tumors (Fig. 2A–B), while patients with lower tumor burden (T1–T3) showed significantly lower PD-L1 compared with controls. Thus, the observed pattern reflects relative differences in PD-L1-associated signal across the heterogeneous circulating large-EV pool rather than a direct measure of tumor-cell PD-L1 abundance. Technical factors may also contribute, as large EV were operationally defined by FSC/SSC parameters and vesicle origin was not assigned; thus, differences in vesicle composition across groups could affect baseline PD-L1 readouts. Further work incorporating vesicle source attribution (e.g., additional lineage markers or molecular profiling) will be required to clarify the underlying biology. Together, these results suggest that PD-L1 expression on circulating large EV reflects tumor burden and systemic immune modulation, underscoring their potential as dynamic immuno-oncologic biomarkers.

The development of second primary malignancies is well recognized in HNSCC, most frequently affecting the head and neck region and the lungs [33]. This pattern likely reflects shared carcinogenic exposures such as tobacco and alcohol use [20]. Pulmonary nodules are frequently detected during follow-up, yet distinguishing metastatic HNSCC from primary lung cancer—particularly squamous cell carcinoma (SQCCL)—remains a major diagnostic challenge with direct therapeutic implications [21]. Molecular analyses, including TP53 mutation profiling, can support this distinction [34, 35], but such assays are time-consuming, require paired tissue samples, and are not routinely available in clinical practice.

In contrast to tissue-based molecular testing, large EV can be obtained from peripheral blood in a minimally invasive manner and analyzed by flow-cytometric immunophenotyping. In our study, combined evaluation of EGFR, EPCAM, and MUC1 expression profiles enabled classification of approximately 75% of HNSCC and NSCLC samples by random forest analysis. This moderate discrimination in a heterogeneous NSCLC cohort supports a disease-associated signal, whereas the stronger performance in the SQCCL-restricted analysis likely reflects increased histologic comparability—but remains limited by the small SQCCL sample size. Restricting the analysis to squamous lung carcinoma (SQCCL) markedly improved discrimination, reaching a sensitivity of 96.9%. Importantly, the random forest model (Fig. 4E) showed a very low classification error for HNSCC (0.046) but a substantially higher error for SQCCL (0.455), indicating that the model is more robust at identifying (‘ruling in’) HNSCC than correctly recognizing SQCCL cases. Consequently, while a positive HNSCC classification may be clinically informative, a non-HNSCC classification does not reliably “rule out” HNSCC in favor of SQCCL and should be interpreted with caution until validated and optimized in larger SQCCL cohorts. These results highlight that large EV surface-marker signatures capture tumor-entity–specific phenotypes, offering a clinically accessible tool for distinguishing HNSCC from primary lung squamous carcinoma.

Despite these encouraging findings, our study has several limitations. Physical and molecular EV characterization was not performed, as the study focused on large EV operationally defined by FSC/SSC parameters and Western Blot. Moreover, because vesicle source attribution (e.g., immune- vs tumor-derived) was not performed, mechanistic interpretation of PD-L1 baseline differences across groups remains limited. Although the HNSCC and NSCLC cohorts were balanced in overall size, the NSCLC cohort was histologically heterogeneous and dominated by adenocarcinoma, while the SQCCL subgroup was small (n = 11; Table 3). Consequently, the HNSCC–SQCCL discrimination shown in Fig. 4 should be interpreted as exploratory, and performance estimates may be unstable; validation in larger, independent SQCCL cohorts is required before clinical translation. An additional limitation is the age imbalance between healthy controls and HNSCC patients (Table 1). Moreover, no cross-validation or independent external validation cohort was available; therefore, all model-based performance estimates should be interpreted as hypothesis-generating. In particular, the HNSCC-versus-control model showed limited specificity at the reported operating point and is therefore not suitable as a standalone clinical classifier in its current form. Since circulating EV and their surface-marker profiles may vary with age, residual age-related confounding cannot be excluded. In our study setting, recruiting age-matched donors meeting strict ‘healthy’ criteria was not feasible; conversely, including non-healthy controls may introduce disease-related alterations in large EV marker expression that are independent of age. Therefore, our findings should be interpreted in the context of an exploratory proof-of-concept study and require confirmation in larger, ideally age-matched cohorts. Moreover, to definitively assess the utility of our panel in distinguishing between pulmonary metastases from HNSCC and second primary lung cancers, future studies should incorporate matched molecular analyses of tissue specimens.

In summary, this study demonstrates the feasibility of flow-cytometric profiling of large EV as a translational liquid biopsy approach in HNSCC. The association between PD-L1 expression on large EV and tumor burden highlights their potential as dynamic biomarkers of disease activity and systemic immune modulation. Moreover, the distinct large EV surface-marker patterns distinguishing HNSCC from SQCCL underscore their diagnostic relevance and support further validation of large EV-based immunophenotyping in larger clinical cohorts.

## Conclusion

In conclusion, this study demonstrates that large EV represent a feasible and clinically accessible platform for immunophenotypic biomarker analysis in HNSCC. PD-L1 expression on large EV correlated with tumor burden, indicating potential utility for disease monitoring and immune status assessment. Taken together, these findings support an exploratory marker panel approach, in which PD-L1 showed the clearest association with tumor burden, whereas EGFR, EPCAM, and MUC1 were most informative in combination rather than as standalone markers. Furthermore, combined evaluation of EGFR, EPCAM, and MUC1 enabled high-sensitivity discrimination between HNSCC and SQCCL, providing a minimally invasive diagnostic adjunct in clinically challenging cases. These findings warrant validation in larger cohorts and integration with tissue-level molecular and immune profiling to confirm their translational applicability.

## Supplementary Information

Below is the link to the electronic supplementary material.

Supplementary file1 (PPTX 1159 kb)

## Data Availability

The datasets generated during and during the current study are available from the corresponding author on reasonable request.
