# Supplementary material for: Flow-cytometric profiling of large extracellular vesicles as immunophenotypic biomarkers in head and neck squamous cell carcinoma
Source: Cancer Immunol Immunother. 2026 May 27;75(6):168. doi: 10.1007/s00262-026-04426-8 (PMC13221563; doi:10.1007/s00262-026-04426-8)
Supplement: Supplementary file 1 — Supplementary file1 (PPTX 1159 kb) [file 262_2026_4426_MOESM1_ESM.pptx]

## Slide 1
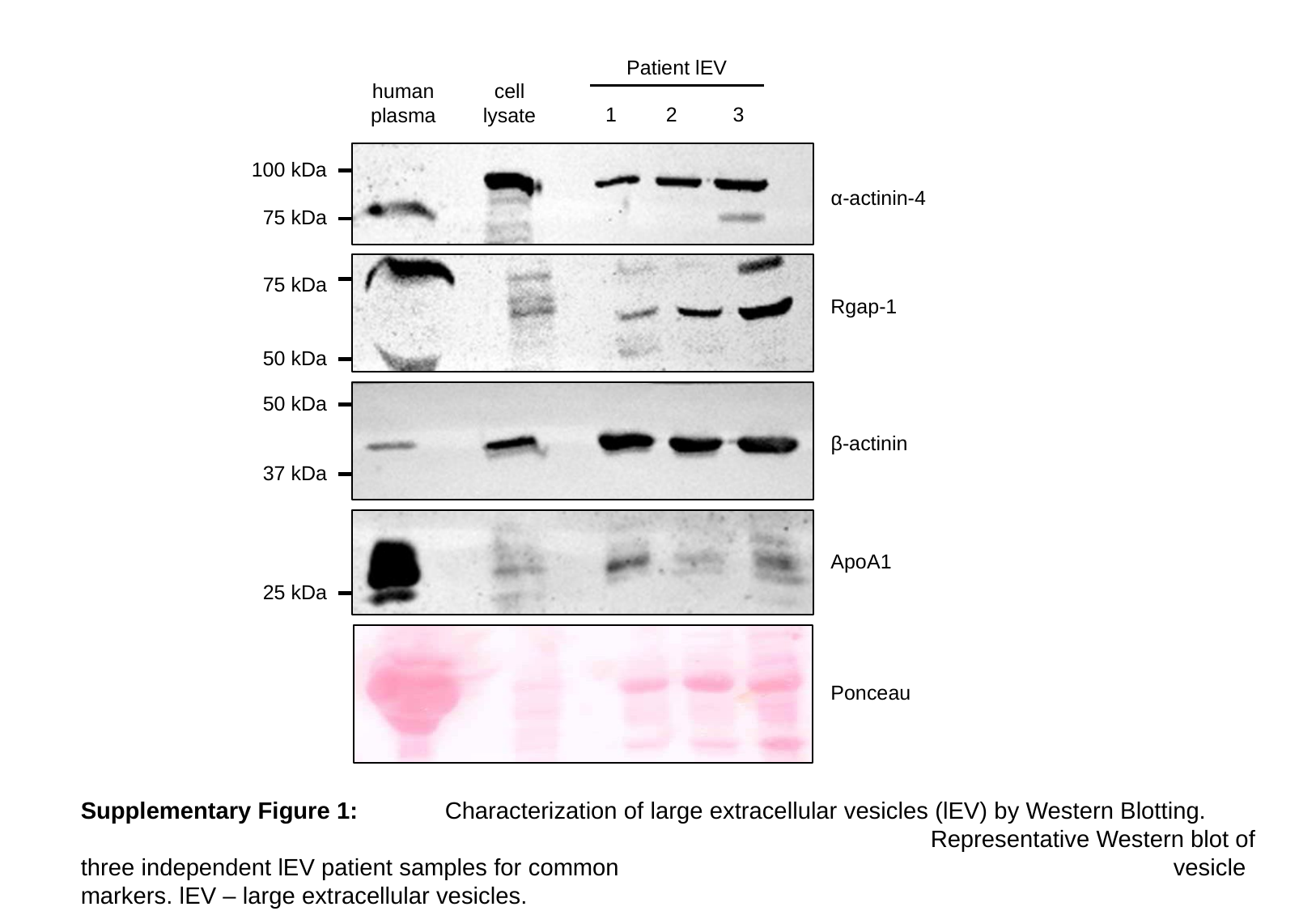

Patient lEV
cell lysate
human plasma
1
2
3
100 kDa
α-actinin-4
75 kDa
75 kDa
Rgap-1
50 kDa
50 kDa
β-actinin
37 kDa
ApoA1
25 kDa
Ponceau
Supplementary Figure 1: 	Characterization of large extracellular vesicles (lEV) by Western Blotting. 							Representative Western blot of three independent lEV patient samples for common 					vesicle markers. lEV – large extracellular vesicles.
